# Anterior Segment Measurement Dataset Using Ultrasound Biomicroscopy Image Analysis

**DOI:** 10.1167/tvst.15.8.19

**Published:** 2026-08-24

**Authors:** Taylor Kolosky, He Eun Forbes, Moran R. Levin, Camilo Martinez, William P. Madigan, Janet L. Alexander

**Affiliations:** 1 University of Maryland School of Medicine, Baltimore, MD, USA; 2 New York Institute of Technology College of Osteopathic Medicine, New York, NY, USA; 3 Department of Ophthalmology and Visual Sciences, University of Maryland School of Medicine, Baltimore, MD, USA; 4 Department of Ophthalmology, Children’s National Hospital, Washington, DC, USA

**Keywords:** ultrasound biomicroscopy (UBM), imaging, pediatric, anterior segment (AS)

## Abstract

**Purpose:**

The purpose of this study was to provide a comprehensive, quantitative dataset of anterior segment (AS) parameters obtained from ultrasound biomicroscopy (UBM) images to support research in ocular development, disease characterization, and image-based analysis.

**Methods:**

UBM images were prospectively collected from 185 eyes of 138 participants aged 3 weeks to 26 years (median = 17 months), encompassing diagnoses such as healthy controls, primary congenital glaucoma (PCG), glaucoma following cataract surgery (GFCS), congenital cataract, traumatic cataract, Lowe syndrome, and Sturge–Weber syndrome (SWS)-associated glaucoma. Twenty-seven quantitative AS parameters were measured from deidentified images using ImageJ software following a standardized protocol.

**Results:**

The resulting dataset includes demographic and diagnostic metadata paired with quantitative UBM-derived parameters for each eye. The dataset is provided in comma-separated value (CSV) format with an accompanying data dictionary.

**Conclusions:**

This dataset provides one of the most extensive collections of quantitative pediatric AS measurements obtained by UBM, enabling characterization of age- and disease-related anatomic variation and facilitating reproducible secondary analyses.

**Translational Relevance:**

This open-source pediatric UBM dataset establishes a foundation for studies of ocular growth, disease mechanisms, and surgical planning, and provides a valuable resource for the development and validation of automated image analysis and machine learning models in pediatric AS imaging.

## Introduction

Ultrasound biomicroscopy (UBM) is a high-frequency imaging technique that provides high-resolution, cross-sectional visualization of the anterior and posterior segment. Unlike optical imaging modalities, UBM can penetrate opaque or edematous tissue, allowing clear visualization of structures such as the ciliary body, iris root, angle, and lens even in the presence of corneal opacity, dense cataract, or active inflammation.^1^ This capability renders UBM especially valuable in pediatric patients, where anterior segment (AS) disease is often accompanied by corneal haze, or other anomalies posterior to the iris.^2^^,^^3^ Additionally, in pediatric ophthalmology, clinical evaluation is often complicated by limited patient cooperation and difficulty obtaining a detailed history. As a result, objective, high-resolution imaging modalities, such as UBM, play a crucial role in accurately characterizing AS anatomy and pathology.

Although UBM has been widely used for qualitative assessment and clinical decision making, quantitative reference data describing AS dimensions in children remain limited. Most available measurements have been derived from adult populations^4^ or optical modalities such as AS optical coherence tomography (AS-OCT),^5^^,^^6^ which are limited in visualizing the posterior chamber and ciliary anatomy. As data-driven approaches and personalized medicine become more widely used, there is increasing need for well-characterized, quantitative datasets that capture normal variation and disease-associated changes across developmental stages, sexes, and racial and ethnic backgrounds.

To address this gap, we constructed a dataset of AS parameters obtained from UBM images of patients across a broad range of ages, demographics, and ophthalmic diagnoses. The dataset includes measurements of key intraocular structures and associated metadata to facilitate reproducibility and secondary analyses. This resource is intended to support research in pediatric ocular development, comparative anatomy, surgical planning, and the development of automated image analysis or machine learning models for AS clinical evaluation.

## Methods

### Participants

All participants were enrolled prospectively with written informed consent for UBM imaging and analysis obtained from the parents, guardians, or participants, depending on age. Enrollment occurred at the time of ocular examination, with most participants imaged during examination under anesthesia (EUA). The remaining participants were imaged awake in the clinic using topical anesthesia. Participants were recruited and imaged at the University of Maryland Medical Center (UMMC) or Children's National Medical Center between November 2014 and May 2025. A total of 185 eyes from 138 participants aged from 3 weeks to 26 years (median = 17 months) were included. Diagnoses represented included healthy controls, primary congenital glaucoma (PCG), glaucoma following cataract surgery (GFCF), Lowe (oculocerebrorenal) syndrome, congenital cataract, traumatic cataract, and Sturge Weber syndrome (SWS)-associated glaucoma.

Healthy controls were participants without a history of intraocular disease or trauma presenting for care of a contralateral eye condition, or ophthalmic indications unrelated to the AS. No control participants had any history of intraocular surgery or traumatic injury to the imaged eye. Contralateral conditions among healthy control participants included trauma, strabismus, ptosis, nasolacrimal duct obstruction, and dermoid cyst. All control participants demonstrated age-appropriate visual acuity and visual behavior, normal comprehensive eye examination, and normal dilated fundus examination by a board-certified pediatric ophthalmologist.

PCG was operationally defined as glaucoma diagnosis prior to the age of 2 years without concurrent AS anomalies, and without history of surgery or trauma. Although this definition differs from the Childhood Glaucoma Research Network classification and World Glaucoma Association consensus statement, no participants in our cohort had isolated primary glaucoma diagnosed after 2 years of age, and therefore this distinction was unlikely to meaningfully affect diagnostic characterization within the study population. GFCS was defined as glaucoma developing after congenital cataract surgery, and cases were further classified based on whether the angle was open or closed. SWS-associated glaucoma was defined as glaucoma diagnosed in a child with a clinical diagnosis of SWS. Glaucoma diagnosis required intraocular pressure (IOP) greater than 21 millimeters of mercury (mm Hg) with one or more of the following structural changes: (1) increased corneal diameter (greater than 11.0 mm before age 1 year or greater than 12.0 mm between age 1 and 2 years), or greater than 1.5 mm of asymmetry of corneal diameter, (2) progressive myopic shift concurrent with an increase in corneal diameter and/or axial length, (3) increased optic nerve cupping by 20% or greater (as measured using cup-to-disc ratio), or (4) the need for surgical intervention for IOP control.

Congenital cataract was defined as a lens opacity that was present since birth or presumed to have developed during infancy, regardless of the age at diagnosis. Cases were classified as congenital when there was no history of trauma or other acquired cause and clinical or historical features (e.g. bilateral involvement or early amblyogenic opacity) supported a developmental or prenatal etiology. Traumatic cataract was defined as a lens opacity secondary to blunt or penetrating ocular injury, confirmed by history or documentation of trauma. Cases were excluded if the cause of lens opacity could not be confidently attributed to trauma.

This study adhered to the ethical principles outlined in the Declaration of Helsinki, as amended in 2013. The institutional review board of the University of Maryland has approved the above referenced protocol and the associated consent forms. Collection and evaluation of protected health information was compliant with the Health Insurance Portability and Accountability Act of 1996.

### Imaging

UBM images were obtained from 185 eyes of 138 patients using either the Aviso Ultrasound Platform A/B UBM with a 50-megahertz (MHz) linear transducer (Quantel Medical, Clermont-Ferrand, France) or the Accutome UBM Plus Platform with 48-MHz linear transducer (Keeler Accutome, Inc., Malvern, PA). Prior studies have shown no significant differences in AS measurements across comparable AS imaging modalities.^7^^–^^9^ All images were acquired by a trained board-certified pediatric ophthalmologist followed the standardized protocol described by Qureshi et al.^10^

This dataset comprises several subsets of images collected for different initial research purposes; therefore, not all image orientations or measurements were available for each eye. Standard orientations included horizontal and vertical cross-sections of the eye at the center of the pupil, four angle images at the intersection of the cornea and iris (angles at the 12, 3, 6, and 9 o'clock positions) centered at the trabecular-iris angle, and one focused corneal image. Imaging tools and conditions included the use of an Afonso eyelid speculum, Hypromellose ophthalmic solution (2.5%), a ClearScan probe cover, and standardized lighting conditions without pharmacologic dilation.

Images were selected based on overall image quality, centration of the pupil, visibility of the angles, with resolution sufficient to localize the scleral spur on each side of the image, and identification of key anatomic landmarks including the corneal center and angles. All images were deidentified to remove PHI (patient name and date of birth [DOB]) and were assigned unique identification codes corresponding to participant, eye, and image orientation.

### Image Analysis

AS parameters were measured directly from UBM images using ImageJ software (National Institutes of Health), a Java-based open access image processing program. The measurement protocol and validation have been previously described by Qureshi et al.^10^ Measurements were obtained by two independent trained observers. To ensure reproducibility, intra-class correlation coefficient (ICC) was calculated for each set of measurements. Only measurements with an ICC ≥0.7 were included, and the final values were averaged between observers.

Not all parameters were available for each eye due to variations in imaging purpose or image orientation; however, the full dataset includes 27 unique parameters (Table 1). The dataset is publicly accessible at https://doi.org/10.5281/zenodo.19599586 under an open-access license, facilitating reproducibility and secondary analyses.

## Data Summary

The final dataset contains 1086 AS measurements of 27 unique structural parameters obtained from UBM image analysis of 185 eyes from 138 participants between the age of 3 weeks and 26 years (median = 17 months) (Supplementary Material). Participant demographics are summarized in Table 2. Forty-seven participants contributed both eyes and 91 participants contributed one eye. Participants are labeled by unique study identification number and specific information related to their age at time of imaging, sex, race, ethnicity, and diagnosis. Each eye also has its own unique identification number. The dataset is available in comma-separated values (CSV) format and is accompanied by a data dictionary describing parameter definitions and variable coding (e.g. sex, race, ethnicity, and diagnosis).

**Table 1.** Structural Parameters

Parameters	Abbreviation, Units	Description
Angle-based measurements		
Angle-angle distance	AA, mm	The linear distance from angle to angle, averaged between horizontal and vertical sections
Angle opening distance	AOD, mm	Perpendicular distance from the inner corneal endothelium 500 µm from TIA apex to outer surface of iris
Angle to scleral spur	A to SS, mm	Linear distance from angle to scleral spur
Scleral spur to anterior ciliary body	SS to anterior CB, mm	Linear distance from scleral spur to anterior ciliary body
Scleral spur to corneal center, linear	SS to CCT, linear, mm	Linear distance from scleral spur to center of cornea
Scleral spur to corneal center, curved	SS to CCT, curved, mm	Curved (along contours) distance from scleral spur to center of cornea
Scleral spur to mid-iris insertion	SS to mid-iris, mm	Linear distance from scleral spur to mid-iris insertion
Endothelial curved surface length	ECSL, mm	Arc length along the inner corneal endothelial surface across cross-section
Anterior chamber		
Anterior chamber depth	ACD, mm	Perpendicular distance from the corneal endothelium to the anterior surface of the lens along the visual axis
Cornea		
Central corneal thickness	CCT, mm	Thickness of the center of the cornea, measured from the anterior epithelial surface to the posterior endothelial surface
Anterior corneal radius of curvature	ACRC, mm	Radius of curvature of the anterior corneal surface, derived from a best-fit sphere on the central cornea
Posterior corneal radius of curvature	PCRC, mm	Radius of curvature of the posterior corneal surface, calculated using a best-fit sphere to the posterior corneal contour
Central corneal integrated pixel density	CCID, arb. units/mm	Sum of pixel values in the central corneal tissue on cross-sectional imaging, representing tissue signal density
Peripheral corneal integrated pixel density	PCID, arb. units/mm	Sum of pixel values in the peripheral corneal tissue on cross-sectional imaging, representing tissue signal density
Scleral corneal integrated pixel density	SCID, arb. units/mm	Sum of pixel values in the scleral tissue adjacent to the limbus on cross-sectional imaging, representing tissue signal density
Corneal endothelial thickness	EndoT, mm	Thickness of the corneal endothelial layer in a cross-section
Corneal epithelial thickness	EpiT, mm	Thickness of the corneal epithelial layer in a cross-section
Mean paracentral (3 mm) corneal thickness	x3T, mm	Average corneal thickness measured at four meridians (12, 3, 6, and 9 o’clock) at a 3-mm diameter zone from the corneal center
Mean peripheral, 6 mm, corneal thickness	×6T, mm	Average corneal thickness measured at four meridians (12, 3, 6, and 9 o’clock) at a 6-mm diameter zone from the corneal center
Angle to central cornea, linear	AA to CCT, linear, mm	Linear distance from the angle to the center of the cornea
Angle to central cornea, curved	AA to CCT, curved, mm	Curved (contour-following) distance from the angle to the center of the cornea
Corneal thickness 0.5 mm from angle	×0.5 mmT, mm	Corneal thickness measured at a 0.5 mm radius from the angle
Corneal thickness 1.0 mm from angle	×1.0 mmT, mm	Corneal thickness measured at a 1.0 mm radius from the angle
Lens		
Lens thickness	LT, mm	Linear distance between the anterior and posterior capsules at the center of the pupil, averaged between horizontal and vertical cross-sections
Iris		
Iris diameter #1	ID1, mm	Iris diameter 1 mm from the iris root
Iris diameter #2	ID2, mm	Iris diameter 2 mm from the iris root
Iris diameter #3	ID3, mm	Iris diameter 3 mm from the iris root

arb, arbitrary; TIA, trabecular iris angle.

**Table 2.** Participant Demographics

Variables	Value
Age, mo	
Mean ± SD	39.4 ± 57.8
Median	20
Range	0–309.4
Race, *N* (%)	
Black	68 (51.5)
White	37 (28.0)
Asian	2 (1.5)
Other	25 (18.9)
Ethnicity, *N* (%)	
Hispanic/Latino	22 (16.7)
Sex, *N* (%)	
Female	72 (54.5)
Diagnosis, *n* (%)	
Healthy control	82 (44.8)
PCG	31 (16.9)
GFCS, angle closure	1 (0.5)
GFCS, open angle	8 (4.4%)
Lowe syndrome	2 (1.1%)
Congenital cataract	55 (30.1%)
Traumatic cataract	3 (1.6%)
SWS glaucoma	1 (0.5%)

GFCS, glaucoma following cataract surgery; *n*, number of eyes; *N*, number of participants; PCG, primary congenital glaucoma; SD, standard deviation; SWS, Sturge Weber syndrome.

## Technical Validation

The dataset underwent both analytical and clinical validation. Analytical validation involved two independent observers, trained in our image analysis protocol, measuring the relevant ocular structures. Reproducibility was assessed using the ICC for agreement between observers on the same measurement parameter. All measurements had an ICC ≥0.7. Final values were averaged between observers. Clinical validation involved confirming that the measured parameters were consistent with established clinical diagnoses, such as PCG, cataract, and other relevant conditions. This step ensured that the dataset reflects clinically meaningful relationships between imaging features and disease states. All images contained in the dataset underwent manual review by a board-certified pediatric ophthalmologist (author J.L.A.) for image quality and clinical validation.

## Discussion

This is an open-source dataset of AS measurements obtained using a validated UBM imaging and analysis protocol, along with corresponding demographic and diagnosis information for each participant. We developed and applied a reproducible, standardized imaging and image analysis protocol to quantify a wide range of AS structures. The dataset includes both healthy controls and eyes with various pathologies across a wide age spectrum, enabling comparative analyses of normal development and disease-related anatomic variation.

Potential applications of this dataset include establishing age-related normative data for AS dimensions in children; characterizing developmental changes in parameters such as lens thickness and angle width; and comparing structural alterations across disease states, including congenital and traumatic cataracts or different forms of pediatric glaucoma. Some work in these areas has already been conducted, but further expansion is possible, and this dataset enables novel analyses.^11^^–^^14^ Representative prior studies from our group that have utilized subsets of these data are summarized in Table 3. These investigations have demonstrated the utility of these types of data for characterizing normal AS development,^15^^,^^16^ identifying disease-specific anatomic differences,^11^^,^^17^ establishing measurement protocols,^16^ and demonstrating secondary applications of the data for image analysis and automation.^18^ Public availability of this dataset allows independent validation and exploration of new hypotheses. The dataset may also inform surgical planning in pediatric cataract and glaucoma surgery by identifying anatomic variations relevant to surgical approach. Beyond clinical applications, these data provide a foundation for developing and validating automated UBM image segmentation and measurement algorithms, cross-modality comparisons with AS-OCT, and longitudinal studies of AS growth or remodeling after surgery, trauma, or treatment.

**Table 3.** Representative Studies Demonstrating Practical Applications of the Dataset

Reference	Study Objective	Cohort	Key Finding
Maripudi et al., 2020^15^	Describe corneal development from infancy to early adulthood	Healthy controls, 0–26 y	Significant age-related corneal structural changes were observed in early childhood, including increases in angle-angle distance and progressive flattening of anterior and posterior curvature
Drechsler et al., 2022^17^	Identify corneal structure differences between congenital glaucoma and controls	Healthy controls and congenital glaucoma, 0–25 y	Significant differences in corneal structure were identified between congenital glaucoma and control eyes, including but not limited to angle-angle distance and scleral spur to central corneal distance
Wei et al., 2024^11^	Determine the association between lens thickness and cataract	Healthy controls and congenital cataracts, 0–5 y	Congenital cataract eyes had significantly thinner lenses compared with controls, with abnormal lens thickness associated with cataract diagnosis
Miglani et al., 2025^16^	Demonstrate reproducible protocol for iris measurements and provide a normative iris thickness dataset by age	Healthy controls, 0–4 y	Iris thickness increased with age, and UBM-based iris measurements demonstrated good reproducibility across imaging conditions and processing approaches
Alexander et al., 2021^18^	Compare semi-automated to manual UBM image analysis of anterior segment structures	Healthy controls and congenital glaucoma, 0–50 y	Semi-automated image analysis demonstrated strong agreement with manual measurements and substantially improved processing efficiency

Previously published AS imaging studies focus on adults^4^^,^^19^ or narrow pediatric subgroups, such as glaucoma or congenital cataracts, whereas few include a broad range of ages and ocular diseases.^6^^,^^20^^,^^21^ In contrast, our dataset uniquely quantifies ciliary body and posterior chamber parameters, which are difficult to visualize with optical modalities and are poorly characterized in the literature, in a population of patients of varying ages and disease states. In addition, pediatric eye imaging is inherently challenging due to smaller anatomy, limited patient cooperation, and the frequent need for examination under anesthesia,^22^ making high-quality datasets such as this particularly valuable. By including standardized metadata such as demographics and diagnostic classifications, this open-access dataset addresses a major gap in reproducible, quantitative pediatric AS imaging and facilitates cross-study and cross-modality comparisons.

Limitations include incomplete parameter availability for all eyes, as imaging was initially performed for varying research purposes, and some patients’ anatomy or cooperation limited acquisition of specific orientations. Images were acquired using two different UBM devices, which may introduce minor inter-device variability. Future work will aim to expand the dataset's size and diversity, incorporate longitudinal follow-up, and perform systematic inter-device comparison studies to enhance reproducibility.

## Conclusions

This open-source pediatric AS dataset represents a valuable resource for both clinical and research applications. By combining high-resolution UBM imaging with standardized measurements and comprehensive metadata, it provides a foundation for advancing our understanding of AS development, disease, and surgical outcomes in children, and for supporting future innovations in automated imaging analysis and multimodal integration.

## Supplementary Material

Supplement 1
